# Cost-effectiveness of public health interventions - a new methodological approach

**DOI:** 10.1186/1472-6963-14-S2-O16

**Published:** 2014-07-07

**Authors:** Inna Feldman, Anna Sarkadi

**Affiliations:** 1 Department of Women’s and Children’s Health, Uppsala University, Uppsala, Sweden

## Background

The goal of public health interventions is to move the distribution of life style risk factors in a healthier direction to decrease disability-causing disease and improve quality of life in the population. We recently presented a novel method to estimate the population-level impact of a public health intervention, using changes in the distribution curve of the outcome of interest. This method makes it possible to calculate the proportion of the target population that benefited from an intervention. The objective of this study is to demonstrate the potential of the new method to estimate not only the effect, but also the cost-effectiveness of public health interventions.

## Method

A population-based model called Risk factors, Health and Societal Costs (RHS) was developed to simulate changes in incidence and related societal costs of several chronic diseases, following assumed changes in four life style risk factors: obesity, tobacco smoking, physical inactivity, and risky consumption of alcohol. The RHS model is based on relative risks and simulates changes in disease incidence due the reduction of the prevalence of a risk factor that can be attributed to diverse interventions. The health gains are calculated as decreased incidence of the disease, increased health-related quality of life years (QALYs) and decreases in disability-adjusted life years (DALYs). Swedish national registers were used to retrieve disease-specific medical care costs, while local authority costs for care and sickness insurance expenses were estimated via a Swedish study. The changes in the distribution of physical inactivity as a result of a hypothetical mass-media campaign targeting the adult population in Uppsala County was used as the input parameter for the model.

## Results

The hypothetical intervention was estimated to improve the distribution of physical inactivity and the overlapping area between the distribution curves at baseline and follow-up was calculated as two percent, i.e. a 2% reduction in the prevalence of physical inactivity. This reduction among the Uppsala County population under five years is estimated to lead to a health gain of 14 QALYs and societal savings (health care, municipality care and sickness insurance) of 6 million SEK (500,000 GBP). The intervention is estimated as cost-effective if the intervention costs are less than 900,000 GBP with ICER<30,000 GBP/QALY.

## Conclusions

This study opens a new avenue to calculate the cost-effectiveness of public health interventions by using changes in the distribution curve of health-related outcomes combined with population-based modeling of risk reductions and costs.

